# Identification of Effective Diagnostic Biomarkers and Immune Cell Infiltration in Atopic Dermatitis by Comprehensive Bioinformatics Analysis

**DOI:** 10.3389/fmolb.2022.917077

**Published:** 2022-07-14

**Authors:** Chenyang Li, Yongping Lu, Xiuping Han

**Affiliations:** ^1^ Department of Dermatology, Shengjing Hospital of China Medical University, Shenyang, China; ^2^ NHC Key Laboratory of Reproductive Health and Medical Genetics, Liaoning Research Institute of Family Planning, The Affiliated Reproductive Hospital of China Medical University, Shenyang, China

**Keywords:** atopic dermatitis, immune cells infiltration, diagnostic biomarkers, bioinformatics analysis, immunohistochemical verification

## Abstract

**Background:** Atopic dermatitis (AD) is a dermatological disorder characterized by symptoms such as chronically inflamed skin and frequently intolerable itching. The mechanism underlying AD development is still unclear. Our study aims to identify the diagnostic and therapeutic biomarkers for AD and provide insight into immune mechanisms at the molecular level through bioinformatics analysis.

**Methods:** The GSE6012, GSE32924, and GSE36842 gene expression profiles were obtained for analysis from the Gene Expression Omnibus database. Differentially expressed genes (DEGs) were segregated using the “Batch correction” and “RobustRankAggreg” methods. Weighted gene co-expression network analysis (WGCNA) was performed to screen for module genes with AD traits. Then, common DEGs (co-DEGs) were screened out via combined differential expression analysis and WGCNA. Functional enrichment analysis was performed for these co-DEGs using Gene Ontology (GO) and the Kyoto Encyclopedia of Genes and Genomes (KEGG), followed by protein-protein interaction network analysis. Candidate hub genes were identified using the “cytoHubba” plugin in Cytoscape, and their value for AD diagnosis was validated using receiver operating characteristic curve analysis in the external database GSE120721. Immunohistochemical staining was performed for further validation. The CIBERSORT algorithm was used to evaluate skin samples obtained from healthy controls (HCs) and lesions of AD patients, to determine the extent of immune cell infiltration. The association between the identified hub genes and significant differential immune cells was analyzed using Pearson correlation analysis.

**Results:** A total of 259 DEGs were acquired from the intersection of DEGs obtained by the two independent procedures, and 331 AD-trait module genes were separated out from the blue module via WGCNA analysis. Then, 169 co-DEGs arising from the intersection of the 259 DEGs and the 331 AD-trait module genes were obtained. We found that co-DEGs were significantly enhanced in the type I interferon and IL-17 signal transduction pathways. Thirteen potential hub genes were identified using Cytoscape. Five hub genes (CCR7, CXCL10, IRF7, MMP1, and RRM2) were identified after screening via external dataset validation and immunohistochemical analysis. We also identified four significant differential immune cells, i.e., activated dendritic cells, plasma cells, resting mast cells, and CD4^+^ naïve T cells, between AD patients and HCs. Moreover, the relationship between the identified hub genes and significant differential immune cells was analyzed. The results showed that the CCR7 expression level was positively correlated with the number of CD4^+^ naïve T cells (R = 0.42, *p* = 0.011).

**Conclusion:** CCR7, CXCL10, IRF7, MMP1, and RRM2 could be potential diagnostic and therapeutic biomarkers for AD. CCR7 expression level was positively correlated with the number of CD4^+^ naïve T cells in AD. These findings need to be corroborated in future studies.

## Introduction

Atopic dermatitis (AD) is a dermatological disorder that presents as chronically inflamed skin and often intolerable itching (Langan et al., 2020). Globally, it is one of the most prevalent skin conditions, affecting approximately 2.1–4.9% of adults and 20% of children (Nutten, 2015; Barbarot et al., 2018). AD causes severe psychological and social hardships and is associated with a high risk of depression, anxiety, work absenteeism, and suicidal tendencies (Eckert et al., 2017; Patel et al., 2019; Sandhu et al., 2019; Schonmann et al., 2020).

The occurrence of AD could be attributed to various factors, including environmental factors, hereditary tendencies, epidermal dysfunction, skin microbiome abnormalities, and immune dysregulation. Immune dysregulation plays a substantial role in the progression of AD (Li et al., 2021). The main immunological mechanisms underlying AD have been attributed to an imbalance of T helper (Th)1/Th2 differentiation. A Th2 cell-mediated response triggers the acute phase, while the chronic phase is triggered by a shift to a Th1 cell-mediated response (Salvati et al., 2021). Damage to the epidermal barrier results in penetration of the skin by antigens, causing the release of alarmins such as interleukin (IL) -25, IL-33, and thymic stromal lymphopoietin (Salimi et al., 2013). These events result in the stimulation of innate type 2 lymphoid cells and inflammatory epidermal dendritic cells. Naïve CD4^+^ T cells also gravitate towards Th2 cells, following the generation of IL-4, IL-5, and IL-13 (Cosmi et al., 2019). In addition to triggering type 2 inflammation, immune cells including Th1, Th17, and mast cells are involved in mixed inflammatory responses (Ho and Kupper, 2019; Abreu and Kim, 2021). However, the immune mechanisms underlying AD have not been described adequately. Investigation of the role played by immune cells and the crucial genes related to associated immune responses needs to be conducted in a methodical manner, and was the focus of the current study.

Several novel genes and biomarkers have been identified for various diseases following the widespread use of bioinformatics analysis techniques and the development of various algorithms. Weighted gene co-expression network analysis (WGCNA) is a sophisticated data processing tool that is widely used to detect clusters or modules of highly correlated genes. It can identify a cluster of genes with similar biological functions, and it can be used to investigate connections between clinical characteristics and gene expression (Langfelder and Horvath, 2008). Besides, an algorithm known as cell-type identification by estimating relative subsets of RNA transcripts (CIBERSORT) has been developed for estimating numbers of immune cells (Chen et al., 2018). This tool has been utilized to measure the extent of immune cell infiltration in various immune-mediated skin disorders, including psoriasis and acne vulgaris (Yang et al., 2021; Zhang et al., 2021).

Here, we aimed to identify diagnostic and therapeutic biomarkers for AD via the analysis of gene expression omnibus (GEO) database using R packages and online bioinformatics tools. We also aimed to determine the pathways related to AD pathogenesis and mechanisms of immune cell infiltration. We believe this is the first study to apply both the WGCNA and CIBERSORT methods for integration of datasets from various platforms to understand the molecular mechanisms involved in AD, and to verify the results via immunohistochemical method. We are confident that this study would provide a new direction to research on AD management.

## Materials and Methods

### Data Acquisition and Workflow

**FIGURE 1:**
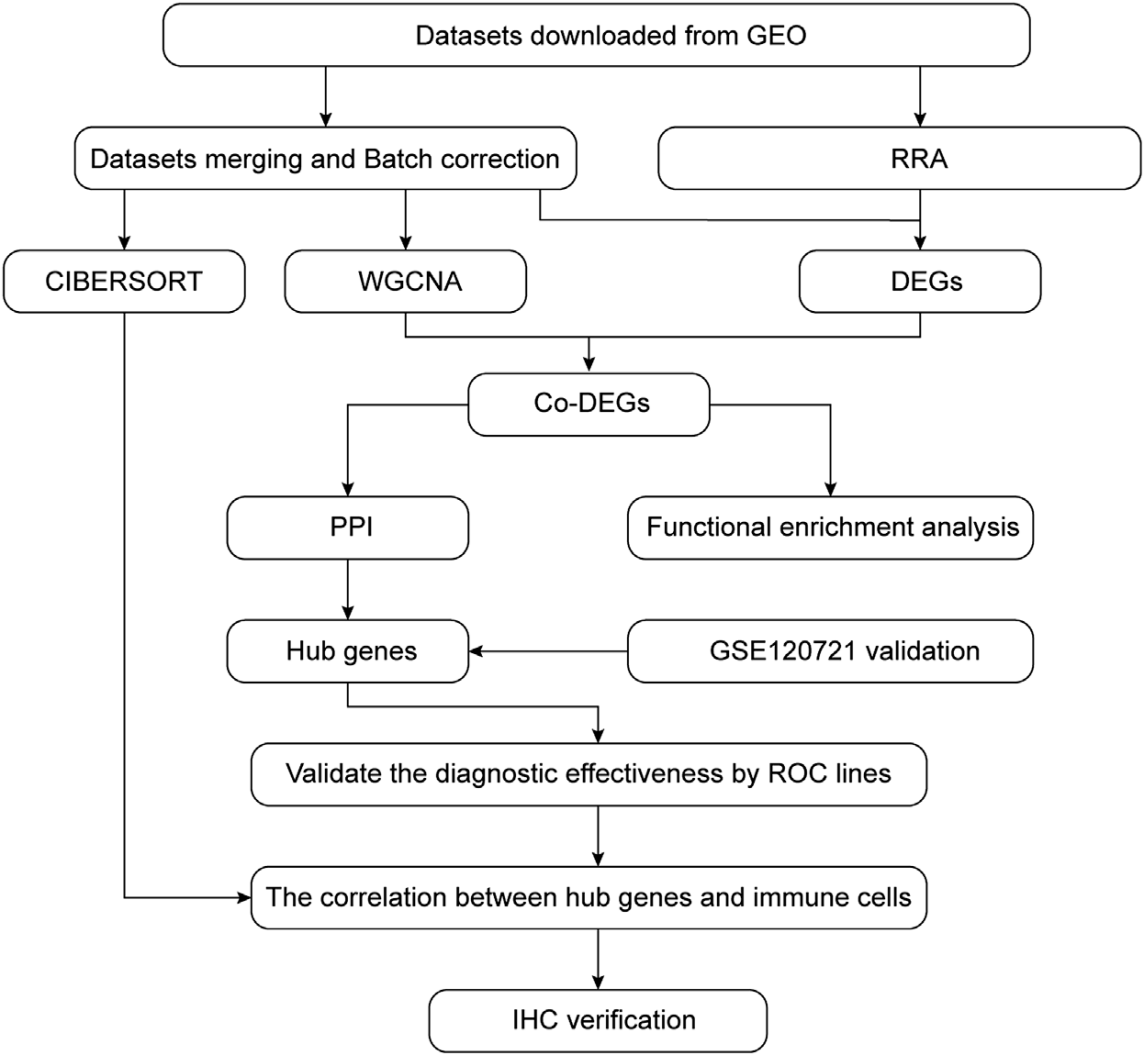
Flow chart summarizing the procedures used in this study.

The GEO database (http://www.ncbi.nlm.nih.gov/geo) was used to segregate gene expression profiles. Gene expression profiles were included if 1) high-throughput sequencing or array-based mRNA expression profiling had been performed; 2) it was possible to obtain lesion skin tissues and healthy skin tissues from AD patients and healthy controls (HCs), respectively; and 3) a minimum of six specimens were included in the dataset. Datasets with drug, placebo, vehicle administration, or other treatments were excluded. Finally, the datasets GSE6012, GSE32924, GSE36842, and GSE120721 were selected for use in our study. The data are summarized in Table 1 and the workflow is depicted in Figure 1.

**TABLE 1:** Baseline information regarding the selected datasets.

GEO datasets	Platform	Samples (Number)			Sample characteristics				Experiment type	Attribute	Author (Reference)
		Total	Patients	Controls	Racial group	Age, y	Lesion phase	Severity			
GSE6012	GPL96	20	10	10	Caucasian	Patients: 40.3 (21–50); Controls: 43 (25–50)	NR	NR	Array	Test	Olsson et al. (2006)
											Mobini et al. (2009)
GSE32924	GPL570	21	13	8	NR	Patients: 38.9 (16–81); Control: NR	Acute exacerbation of chronic disease	Moderate-to-severe	Array	Test	Suárez-Fariñas et al. (2011)
GSE36842	GPL570	31	16	15	NR	Patients: 44 (20–67); Control: NR	Acute and chronic	Moderate-to-severe	Array	Test	Gittler et al. (2012)
GSE120721	GPL570	37	15	22	NR	Patients: 39.4 (27–59); Control: NR	Chronic	Moderate-to-severe	Array	Validation	Esaki et al. (2015)

NR, not reported.

### Data Pre-processing

Series matrix files and platform annotation information were downloaded for three microarray datasets, namely, GSE6012, GSE32924, and GSE36842. The three-probe expression matrixes were converted into gene expression matrixes using Perl script. The average value was derived if there was more than one probe corresponding to a gene. Batch effects were then eliminated using the R package “sva.” Thus, a merged, normalized gene expression matrix was obtained for further analysis. The gene expression matrix for the external validation dataset GSE120721 was obtained in a similar manner.

### Construction of WGCNA

The “WGCNA” R package was used to derive modules identified with clinical characteristics, and to identify hub genes. The merged gene matrix was checked to eliminate anomalous specimens which might have escaped from clustering of samples. The topological overlap matrix (TOM) was derived from the adjacency matrix. Genes were divided into modules based on their dissimilarity in TOM. The cut height, minimal module size, and soft-thresholding power were set at 0.25, 30, and 4 (scale-free *R* ^2^ = 0.85), respectively. The absolute value of the correlation coefficient between traits and genes was used to determine the relationship between clinical traits and module eigengenes. A gene was considered to be significant based on the absolute correlation coefficient between traits and genes. A gene was considered to be a member of a module based on its association with certain expression profiles. Genes with a gene significance (GS) > 0.2 and module membership (MM) > 0.8 in the module most relevant to clinical traits were considered as module genes with AD traits (Hu et al., 2020).

### Screening for Differentially Expressed Genes

Two independent methods were used to obtain the most robust differentially expressed genes (DEGs) from several microarray cohorts. In the first approach (“Batch correction”), the three downloaded raw datasets were combined into an expression matrix after the elimination of batch effects and normalization. Then, DEGs were analyzed using the R package “limma”. In the second approach (“RobustRankAggreg, RRA”), the R package “limma” was used to analyze the DEGs of the downloaded gene matrixes. The DEGs from each such derived dataset were then combined using the R package “RRA” (Zhou et al., 2021). The final DEGs identified by the two methods were determined from their intersection using a Venn diagram. A |log fold change| > 1 was set along with an adjusted *p* value <0.05, as the threshold points for DEGs.

### Functional Annotation and Pathway Enrichment Analysis

The gene from the module of interest and the final DEGs obtained by the two methods were intersected to identify the common DEGs (co-DEGs) using a Venn diagram. Then, the co-DEGs were subjected to functional enrichment analysis. Gene names to gene ID conversion of the co-DEGs was achieved using the R package “org.Hs.eg.db.” Gene Ontology (GO) and Kyoto Encyclopedia of Genes and Genomes (KEGG) analysis for co-DEGs was conducted using the R package “clusterProfiler”. Metascape (http://metascape.org/gp/index.html) was then used for comprehensive data analysis. Significance was set at a *p* value <0.05 and q value <0.05.

### Identification and Validation of Hub Genes

The protein-protein interaction (PPI) network for the co-DEGs was constructed using the STRING tool (https://string-db.org/). The interaction file was downloaded and each node gene was scored by 12 algorithms (Maximal Clique Centrality (MCC), Density of Maximum Neighborhood Component (DMNC), Maximum Neighborhood Component (MNC), Degree, Edge Percolated Component (EPC), BottleNeck, EcCentricity, Closeness, Radiality, Betweenness, Stress, and Clustering Coefficient), using the “cytoHubba” plugin of Cytoscape (v 3.7.2). Candidate hub genes were identified from the genes at the junction of these 12 algorithms, then visualized using the R package “UpSet”. The preciseness of these genes was evaluated in the gene expression profile GSE120721 by ROC curve analysis using the R package “pROC”. Hub genes were validated with an area under the curve (AUC) criterion of >0.8 and a differential expression level significance of *p* < 0.05 using the Wilcoxon test (Sun et al., 2021).

### Evaluation of Immune Cell Infiltration and Correlation Analysis Between Hub Genes and Immune Cells

The immune cell proportions were determined using the CIBERSORT R package (http://cibersort.stanford.edu/download.php), with data from the normalized gene expression matrix of AD patients and HCs and the data from expressed reference signature genes (LM22, http://cibersort.stanford.edu/download.php) (Zhang et al., 2021). A violin plot was used to visualize the distribution of these immune cells using the “vioplot” package in R software. A significance criterion of *p* < 0.05 was used for this calculation. The correlation between the identified hub genes and the infiltrating immune cells was conducted by using Pearson correlation analysis. The resulting relationships were studied using the “ggplot2” and “ggpubr” packages.

### Patient Recruitment

The study proposal was reviewed and approved by the Ethics Committee of Shengjing Hospital of China Medical University. AD patients and HCs were included in the study after obtaining written informed consent. AD was diagnosed as per the Hanifin-Rajka criteria (Hanifin and Rajka, 1980). Full thickness skin samples were collected from 12 individuals. These included six AD patients and six HCs matched for age, sex, and race. Details of the study subjects are shown in Supplementary Table S1.

### Immunohistochemical Verification

Extracted skin tissues were fixed with 4% formaldehyde buffer, and 4-μm-thick sections were obtained from paraffinized specimens. Tissue sections were incubated at 60°C for 2 h before the dewaxing process. For antigen retrieval, the sections were autoclaved in a citric acid buffer (pH 6.0) at 115°C for 3 min and quenched in 0.3% H2O2 for 15 min for endogenous peroxidase activity. Then, sections were treated with goat serum, which was used as a blocking solution, for 45 min, and incubated overnight at 4°C with primary antibodies against CCR7 (Abmart, dilution 1:100), CCNA2 (Abmart, dilution 1:200), CXCL10 (Abmart, dilution 1:200), IRF7 (Abmart, dilution 1:100), ISG15 (Abmart, dilution 1:100), MKI67 (Abmart, dilution 1:100), MMP1 (Abmart, dilution 1:200), NCAPG (Abmart, dilution 1:200), and RRM2 (Abmart, dilution 1:200). These sections were treated with the goat anti-rabbit secondary antibody for 30 min at room temperature. Then, 3,3′-diaminobenzidine was used to visualize protein expression and a Nikon Eclipse 80i microscope (Nikon Corporation) was used to capture images. The public domain program “Image-Pro Plus 6.0” was used to measure the immunohistochemical integral optical density (IOD). Sum IODs and areas of each photo were measured, then average optical densities in different groups were compared.

### Statistical Analysis

R studio (version 4.0.3) was used for the analysis of data and GraphPad Prism 7 software (version 7.0, San Diego, CA, United States) was used for the generation of images. Immunohistochemical data were assessed using the Student’s *t-*test. *p* < 0.05 was considered statistically significant.

## Results

### Identification of DEGs

**FIGURE 2:**
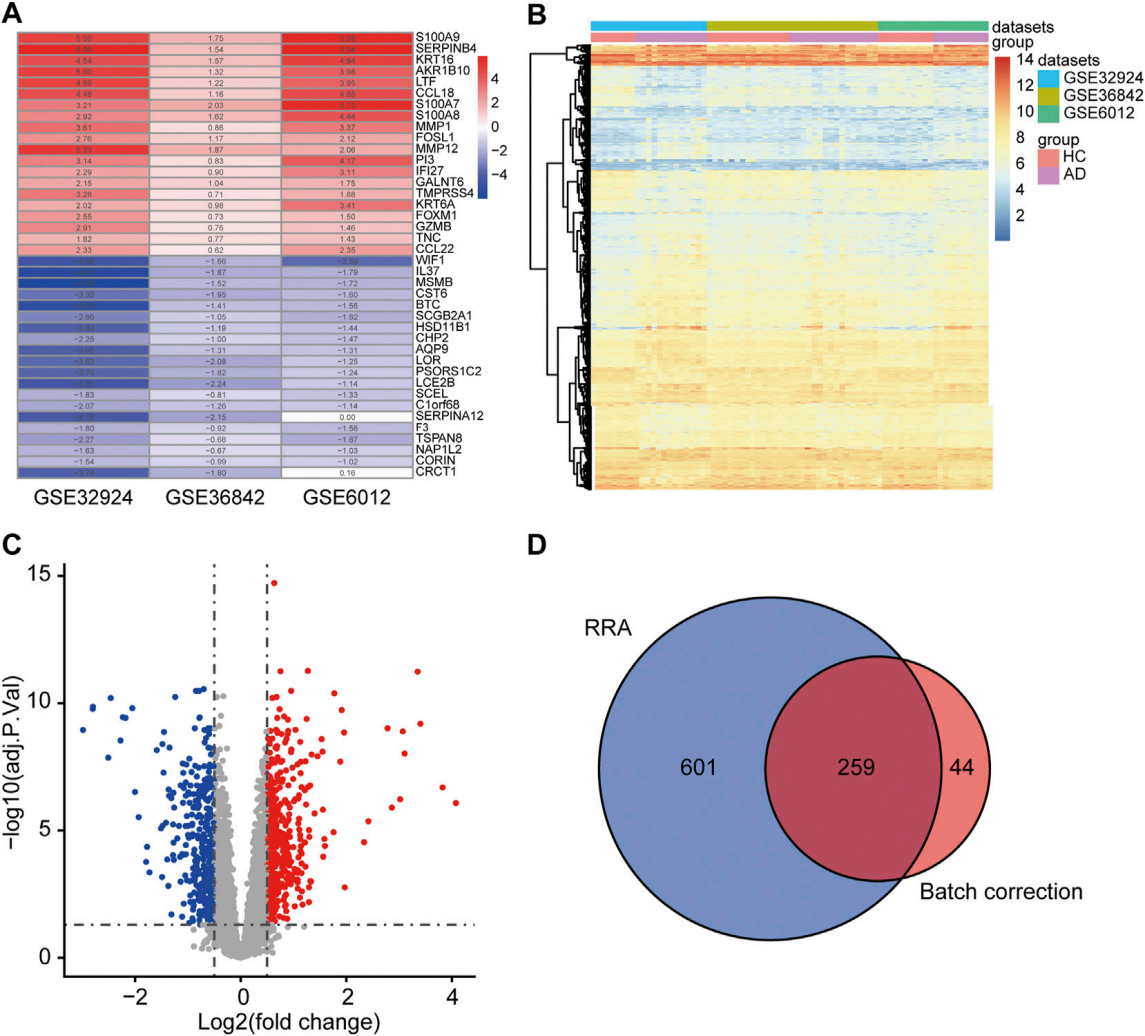
Determination of DEGs. **(A)** The first 20 upregulated and downregulated DEGs of the three datasets merged using the “RRA” approach. **(B)** Heat map of the DEGs obtained via the “Batch correction” approach. **(C)** Volcano map of the DEGs obtained via the “Batch correction” approach. **(D)** Venn diagram of the intersection of the DEGs screened using the two methods. DEGs, differentially expressed genes; RRA, RobustRankAggreg.

DEGs were identified by two methods using three microarray datasets, including 39 AD and 33 HC samples. A total of 303 DEGs were acquired by “RRA.” These consisted of 139 upregulated and 164 downregulated genes, some of which are shown in Figure 2A. A total of 860 DEGs were obtained by “Batch correction.” These consisted of 478 upregulated genes and 382 downregulated genes, which were visualized via a heatmap (Figure 2B) and a volcano map (Figure 2C). A total of 259 DEGs, including 130 upregulated genes and 129 downregulated genes, were acquired from the intersection of the DEGs obtained by the two procedures (Figure 2D and Supplementary Table S2).

### WGCNA and Module Analysis

**FIGURE 3:**
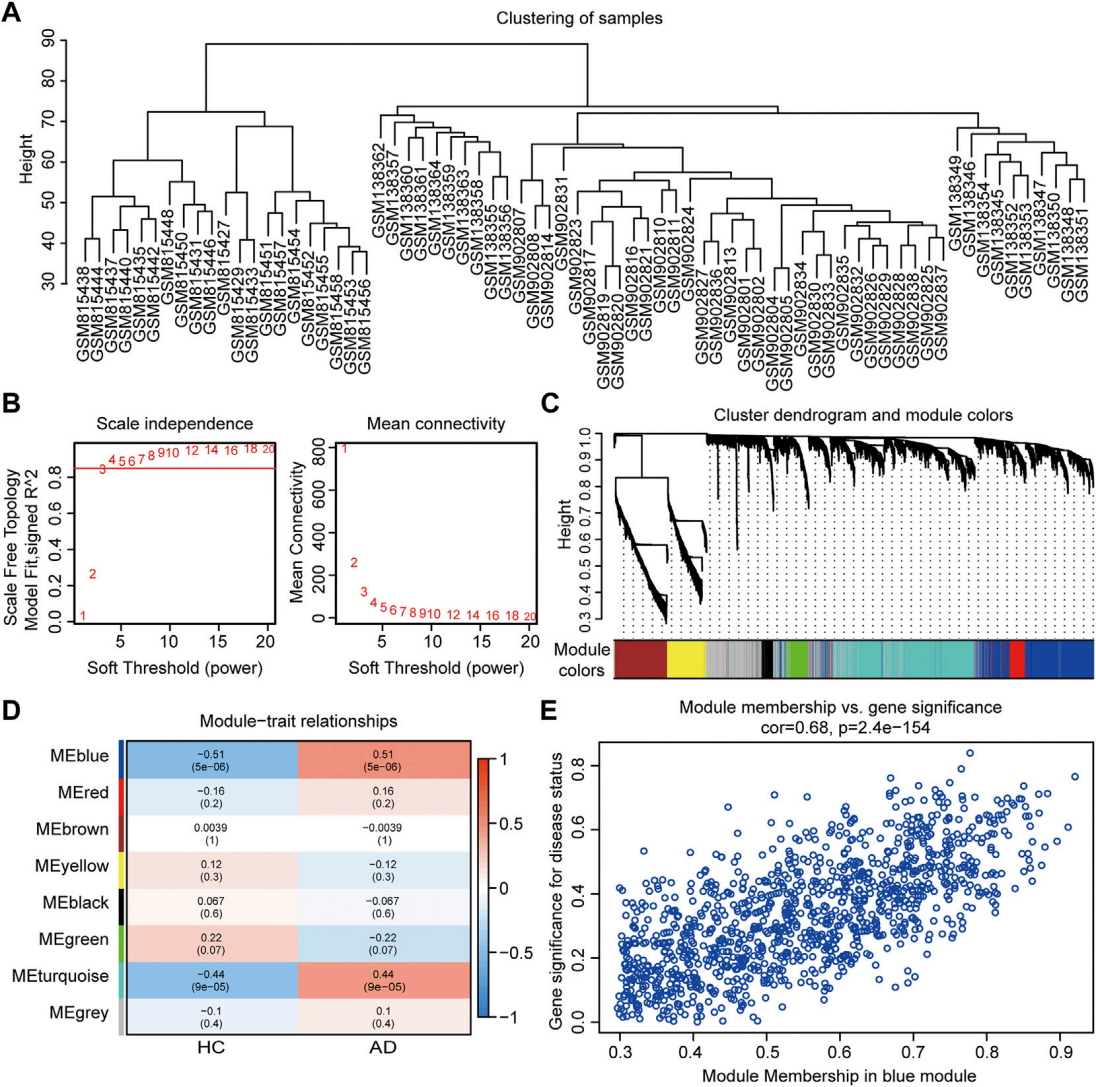
Co-expression network assessment using WGCNA. **(A)** Clustering dendrograms of DEGs from “Batch correction.” **(B)** Assessment of the scale-free fitness index and mean connectivity for various soft-thresholding powers. **(C)** Dendrogram of all the genes clustered based on dissimilarity in the topological overlap. **(D)** Heatmap exhibiting the relationship between module eigengenes and clinical traits. Each row represents a color module, and every column represents a clinical trait. The correlation coefficient and corresponding *p* value are shown in each cell. **(E)** Scatter plot exhibiting genes in the blue module. WGCNA, weighted gene co-expression network analysis; DEGs, differentially expressed genes.

In WGCNA analysis, the merged gene matrix from “Batch correction” was clustered to exclude outliers and abnormal samples which might have escaped during sample clustering (Figure 3A). The soft-thresholding power β was set to 4 to attain a scale-free network evaluation coefficient *R* ^2^ of 0.85 (Figure 3B). Similar modules from the co-expression network were combined using a cut height of 0.25 to obtain a total of eight modules (Figure 3C). Correlations between clinical traits and module eigengenes are shown in Figure 3D. The genes of blue module were determined to be the most relevant to AD, based on the module-trait relationship heatmap (correlation coefficient 0.51, *p* < 0.001). The MM vs. GS for AD in blue module were calculated and presented with a scatter diagram (Figure 3E). Finally, 331 module genes with AD traits from the blue module were identified for further studies (Supplementary Table S3).

### Identification of Co-DEGs and Functional Enrichment Analysis

**FIGURE 4:**
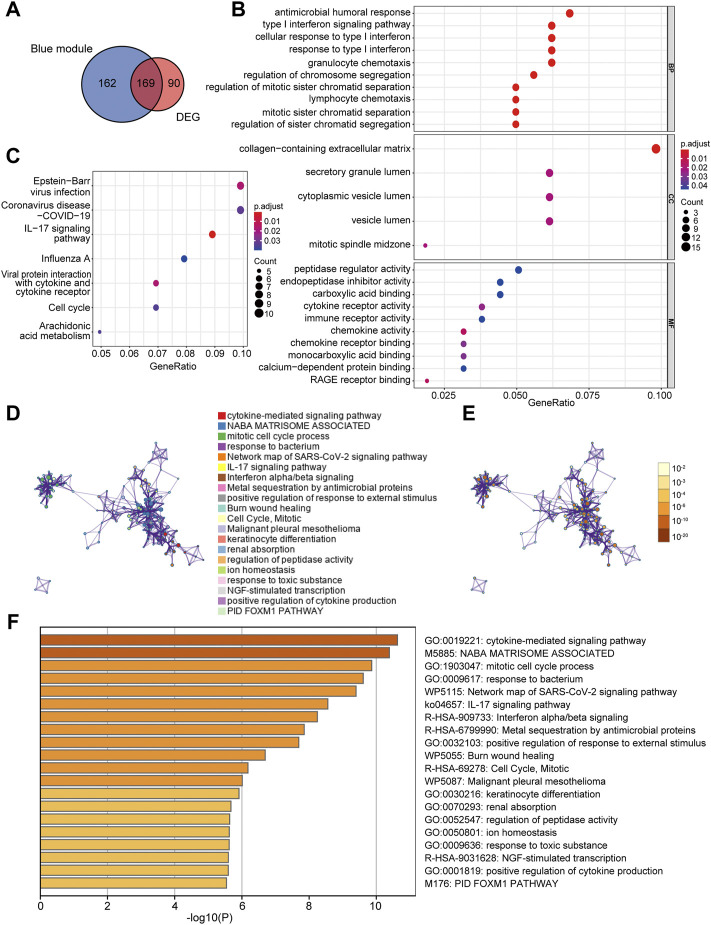
Determination of co-DEGs and functional enrichment analysis. **(A)** Venn diagram illustrating the co-DEGs screened from the intersection of the DEGs and the module genes with AD traits. **(B)** Remarkably enriched GO terms of co-DEGs. **(C)** Remarkably enriched KEGG cascades of co-DEGs. **(D)** Network illustrating terms occurring abundantly in co-DEGs. Every node designates an enriched term colored based on its cluster identity. **(E)** The same enriched network with nodes colored via *p*-value. **(F)** The top 20 pathways linked to co-DEGs based on a comprehensive enrichment assessment. co-DEGs, common differentially expressed genes; AD, atopic dermatitis; GO, gene ontology; KEGG, Kyoto encyclopedia of genes and genomes.

A total of 169 co-DEGs were identified from the intersection of the 331 AD traits module genes and 259 DEGs (Figure 4A). GO and KEGG pathway analyses were used to further investigate the biological properties of these co-DEGs. We identified several biological properties, including cellular components (CCs), biological processes (BPs), and molecular functions (MFs), using GO annotation enrichment terms. With regard to BPs, the co-DEGs were substantially enhanced in the cellular response to type I interferon (IFN) and the type I IFN signaling pathway. As for CC and MF, co-DEGs were substantially enhanced in the collagen-containing extracellular matrix and peptidase regulator activation, respectively (Figure 4B). We identified critical signaling pathways using KEGG enrichment analysis. The co-DEGs were substantially enhanced in the IL-17 signaling pathway (Figure 4C). Metascape analysis indicated that co-DEGs were substantially enhanced in the cytokine-mediated signaling pathway (Figures 4D–F).

### Identification and Validation of Hub Genes

**FIGURE 5:**
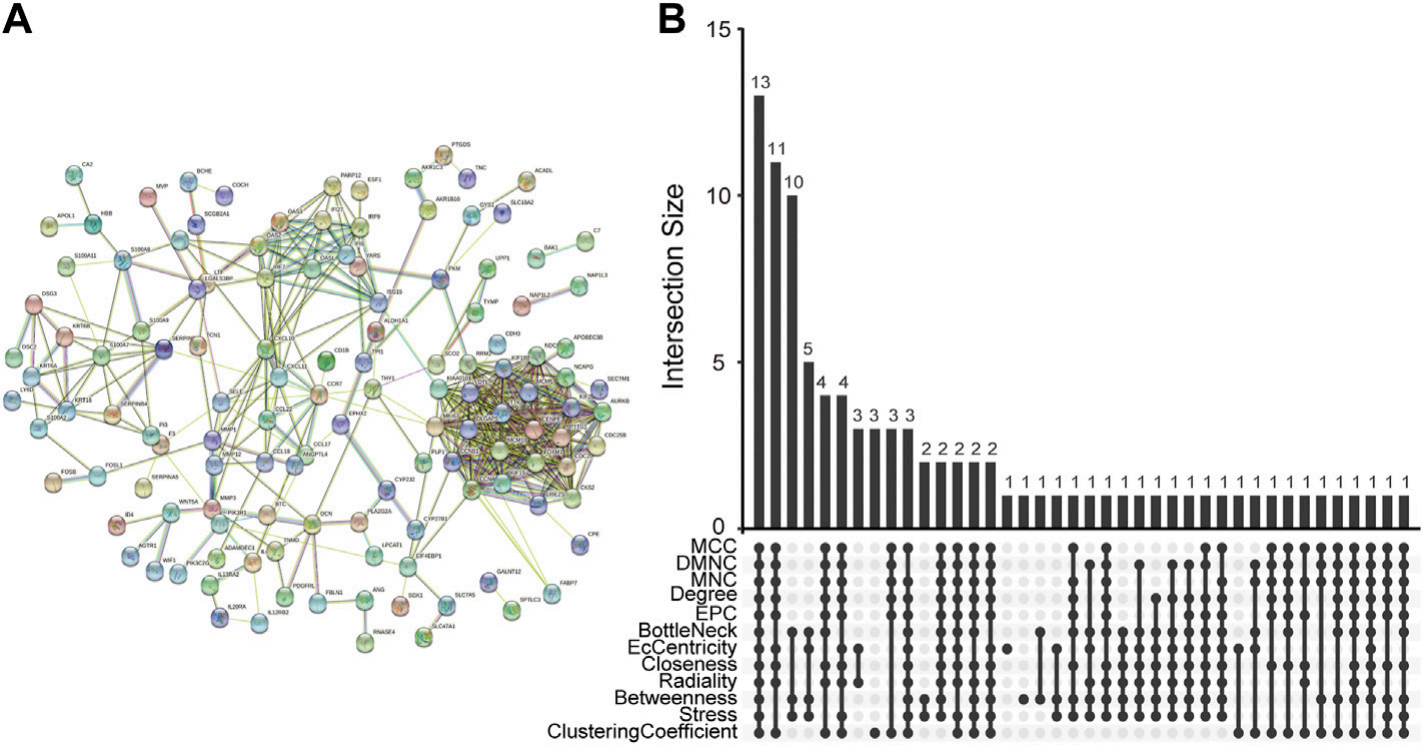
Determination of candidate hub genes. **(A)** The PPI network of co-DEGs. **(B)** Candidate hub genes were screened out using 12 algorithms. PPI, protein-protein interaction; co-DEGs, common differentially expressed genes.

**FIGURE 6:**
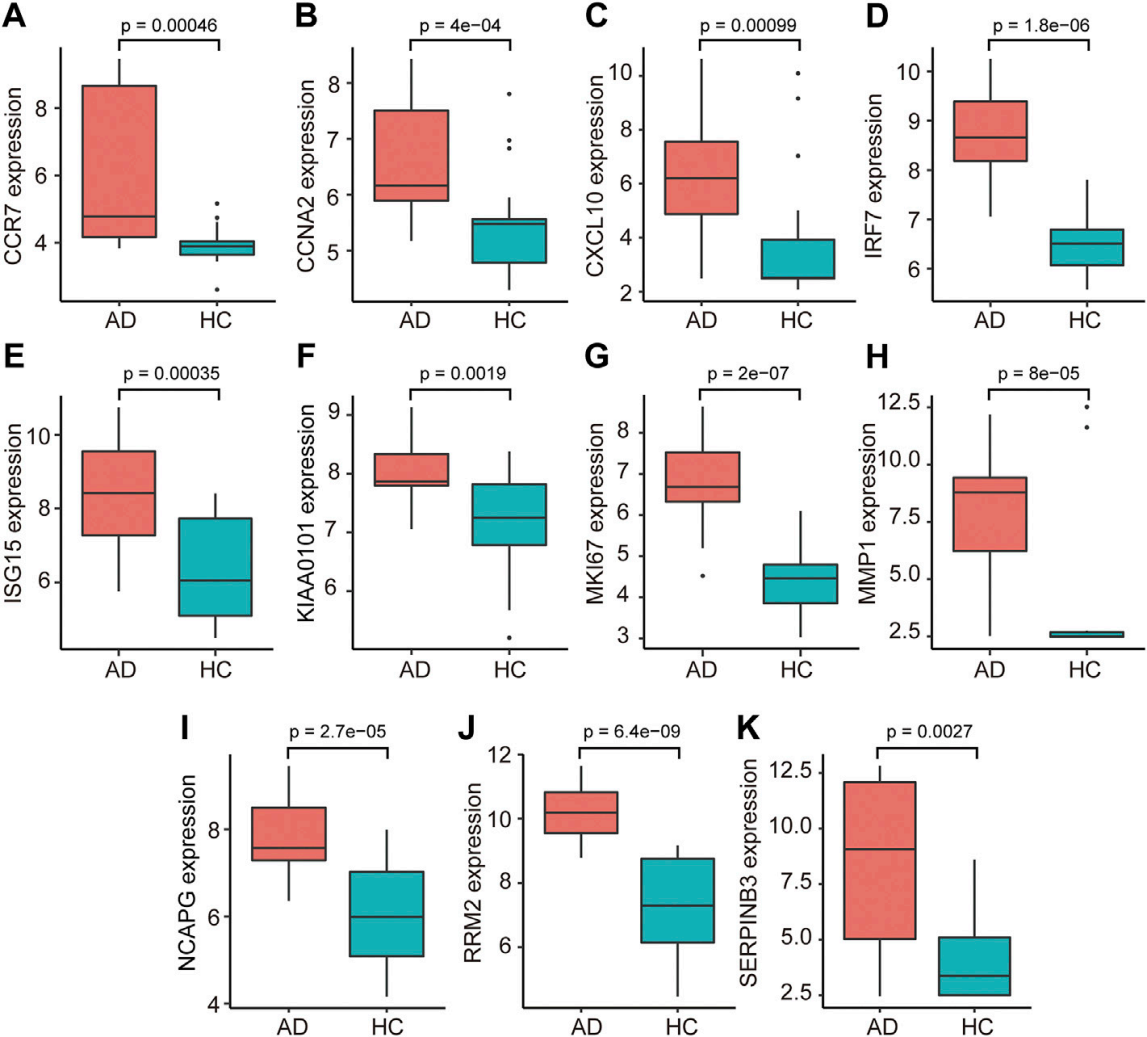
The relative expression levels of 11 candidate hub genes, i.e., (**(A)** CCR7, **(B)** CCNA2, **(C)** CXCL10, **(D)** IRF7, **(E)** ISG15, **(F)** KIAA0101, **(G)** MKI67, **(H)** MMP1, **(I)** NCAPG, **(J)** RRM2, and **(K)** SERPINB3, validated using GSE120721. AD, atopic dermatitis; HC, healthy control.

**FIGURE 7:**
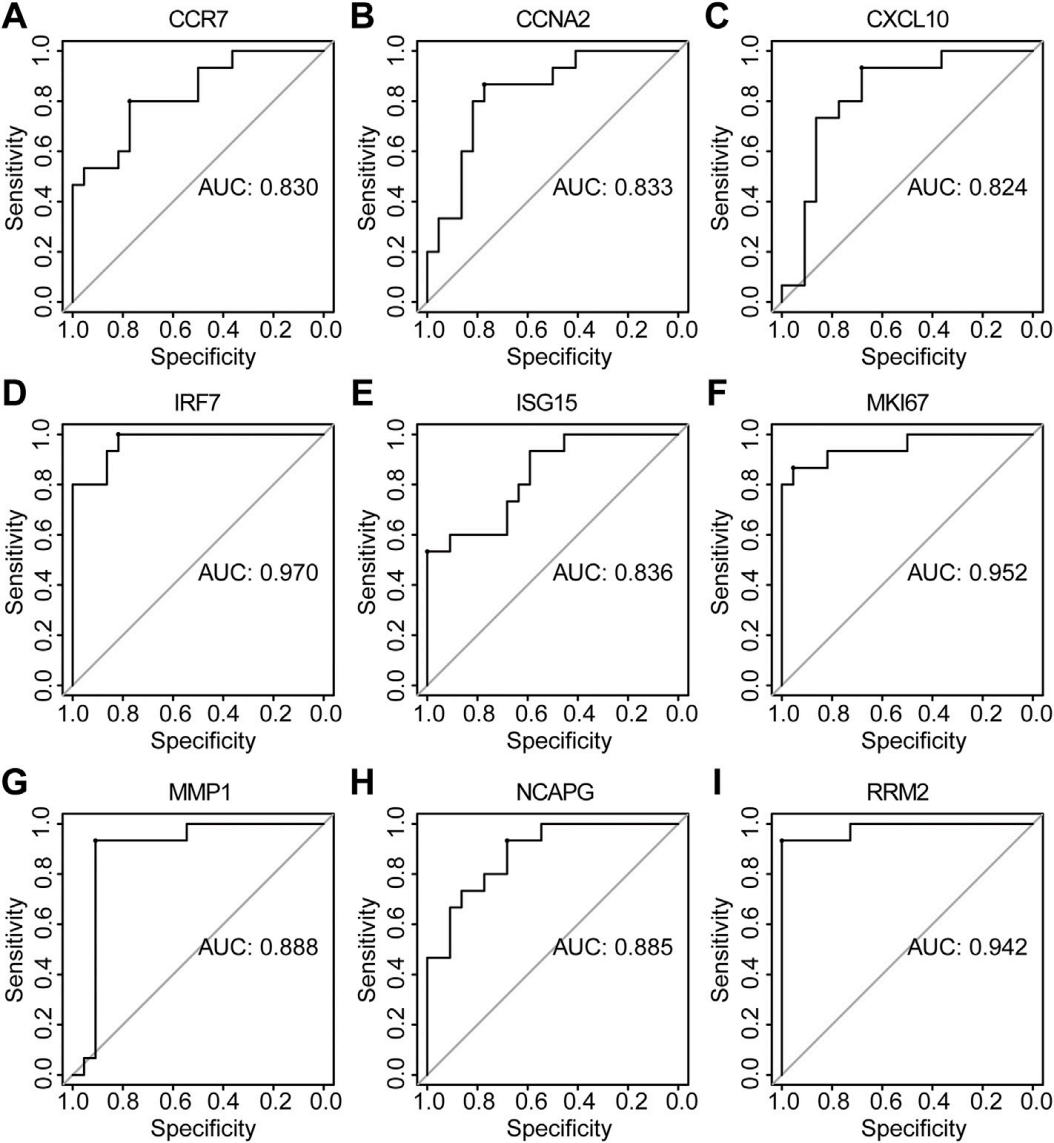
The diagnostic effectiveness of nine hub genes (**(A)** CCR7, **(B)** CCNA2, **(C)** CXCL10, **(D)** IRF7, **(E)** ISG15, **(F)** MKI67, **(G)** MMP1, **(H)** NCAPG, and **(I)** RRM2 was validated using GSE120721.

The PPI network of co-DEGs was constructed by STRING as shown in Figure 5A. 12 algorithms were used to calculate the score of each node gene. Then, 13 candidate hub genes were segregated, including CCR7, CCNA2, CXCL10, CXCL11, IRF7, ISG15, KIAA0101, MKI67, MMP1, NCAPG, RRM2, SELE, and SERPINB3 (Figure 5B). To make the result more reliable, GSE120721 was employed to validate these candidate hub genes. The expression levels of 11 candidate hub genes, including CCR7, CCNA2, CXCL10, IRF7, ISG15, KIAA0101, MKI67, MMP1, NCAPG, RRM2, and SERPINB3, were significantly higher in AD patients (Figure 6). These 11 candidate hub genes were subjected to ROC analysis. An AUC >0.8 was considered indicative of AD, with excellent specificity and sensitivity. Nine genes (CCR7, CCNA2, CXCL10, IRF7, ISG15, MKI67, MMP1, NCAPG, and RRM2) with an AUC >0.8 and significantly different expression levels (*p* < 0.05 as determined by the Wilcoxon test) were identified as hub genes. Among these, IRF7 exhibited the best specificity and sensitivity for the diagnosis of AD (AUC = 0.97) (Figure 7).

### Immunohistochemical Analysis

**FIGURE 8:**
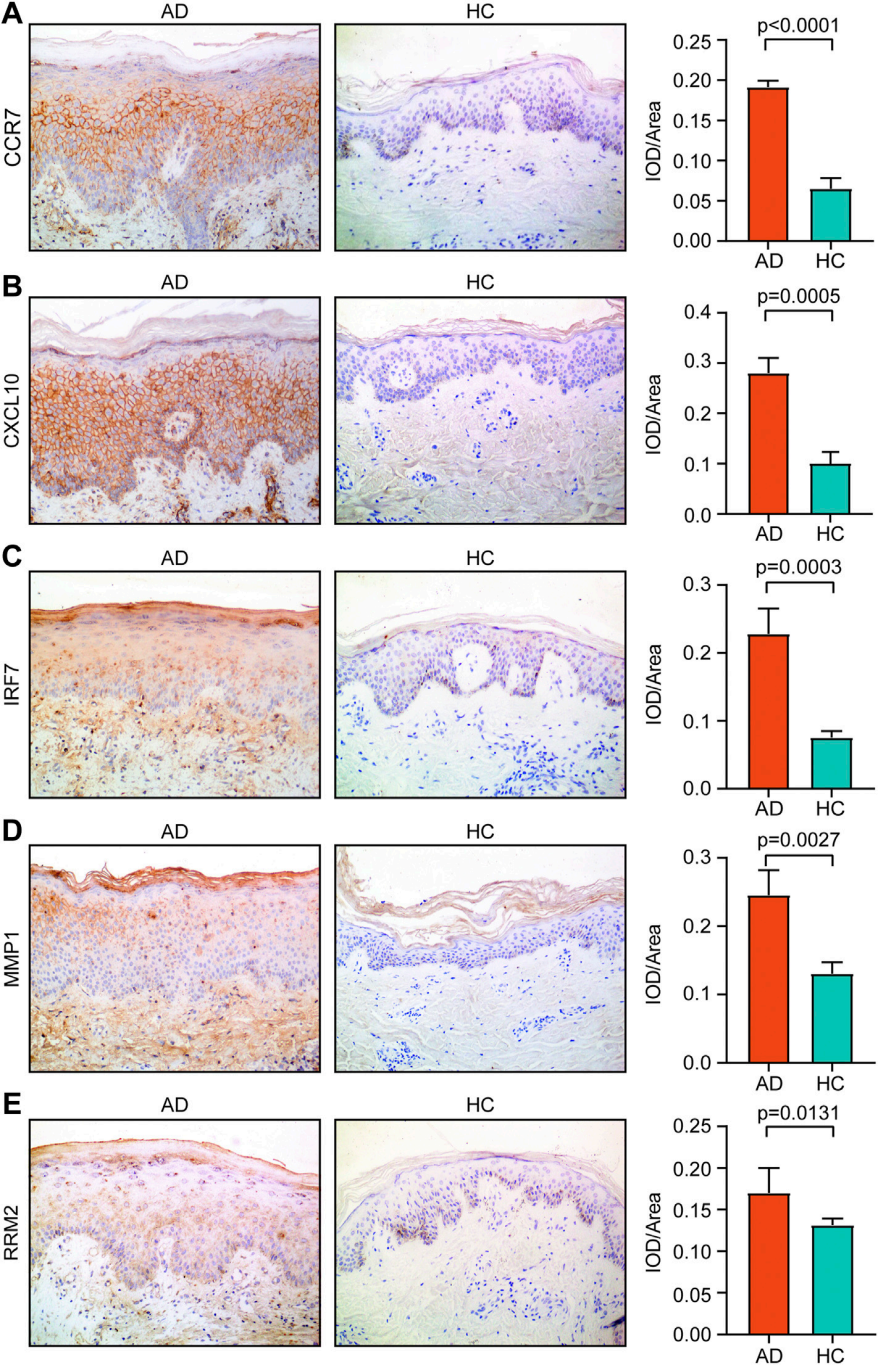
Expression of **(A)** CCR7, **(B)** CXCL10, **(C)** IRF7, **(D)** MMP1, and **(E)** RRM2 in AD tissue and healthy tissue. Magnification, ×200. AD, atopic dermatitis; HC, healthy control.

We found that the IOD/area values of CCR7, CXCL10, IRF7, MMP1, and RRM2 in AD tissue were significantly higher than those in HC tissue (*p* < 0.05) (Figure 8). However, the IOD/area values of CCNA2, ISG15, MKI67, and NCAPG were comparable between the AD and HC tissues (Supplementary Figure S1).

### Ananalysis of Immune Cell Infiltration and the Relationship Between Identified Hub Genes and Differential Immune Cells in AD

**FIGURE 9:**
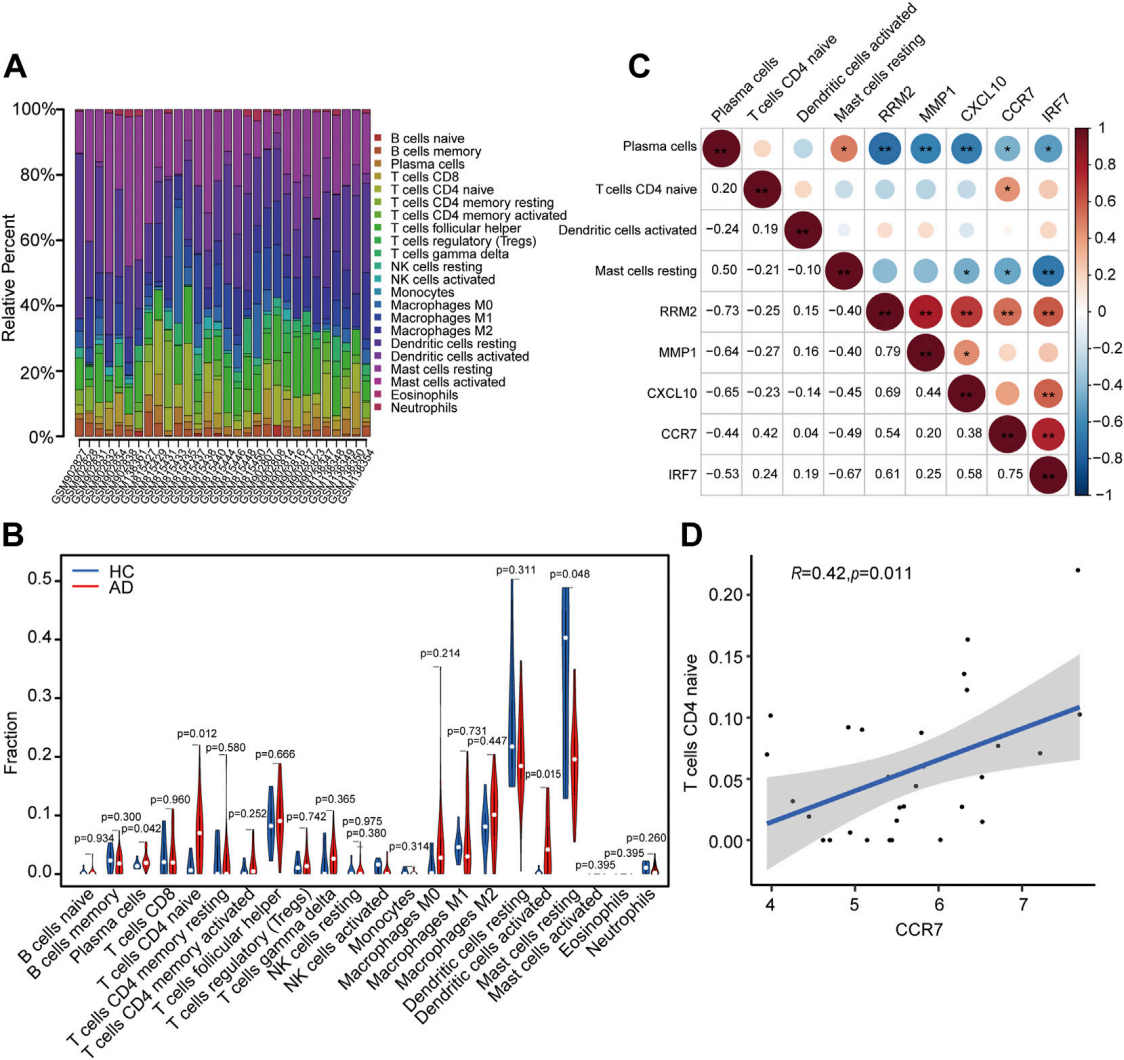
Analysis of immune cell infiltration and the relationship of hub genes with differential immune cells in individuals with AD. **(A)** Relative fraction of 22 sub-populations of immune cells in AD samples. **(B)** The differences of 22 sub-populations of immune cells among the AD and HC tissues. **(C)** Correlation among four remarkable differential immune cells and five identified hub genes. **(D)** Remarkably related hub genes and immune cells screened by the criteria R > 0.4 and *p* < 0.05.

A total of 23 AD and seven HC tissues met the criteria for analysis with CIBERSORT (*p* < 0.05). The constitutions of 22 types of immune cells in each specimen are shown in Figure 9A. Compared with HC tissues, AD tissues exhibited a higher number of activated dendritic cells (DCs), CD4^+^ naïve T cells, and plasma cells, whereas the number of resting mast cells was relatively lower (Figure 9B). Relationships between five identified hub genes (CCR7, CXCL10, IRF7, MMP1, and RRM2) and four significant differential immune cells (naive CD4^+^ T cells, plasma cells, activated DCs, and resting mast cells) in AD tissue were analyzed (Figure 9C). Significantly related hub genes and immune cells were segregated using thresholds of R > 0.4 and *p* < 0.05. We found that the CCR7 expression level was positively correlated with the number of CD4^+^ naïve T cells (R = 0.42, *p* = 0.011) (Figure 9D).

## Discussion

Though research into the molecular processes involved in AD has made rapid advances, the mechanisms of pathogenesis and progression of this disease are still poorly understood, which makes the diagnosis and treatment of this disease a challenge. In this study, we methodically segregated potential new biomarkers for AD and evaluated the extent of infiltration of the skin tissue by immune cells in AD patients. We screened DEGs using two independent methods to improve the precision of the results. We also screened 169 final co-DEGs in combination with WGCNA and differential expression analysis. The results of GO and KEGG analyses showed that co-DEGs were substantially enhanced in the type I IFN and IL-17 signaling pathways.

Multiple IFNα isotypes and IFNβ of the type I family have been extensively studied. Type I IFNs can activate crucial processes of innate and adaptive immune systems, including the presentation of antigens and the production of cytokines responsible for triggering the activation of B cells, T cells, and natural killer cells. These contribute substantially to the pathogenesis of AD (Kader et al., 2021; Li et al., 2021). IL-17 is consistently upregulated in AD in both the acute and chronic phases (Gittler et al., 2012). It may promote abnormalities in immune regulation in AD by upregulating S100A7/8/9 in combination with IL-22 (Nograles et al., 2008). The S100A proteins are highly upregulated in AD, and can operate as both promoters of inflammation and as antimicrobials (Mansouri and Guttman-Yassky, 2015). It has been reported that IL-17 downregulated filaggrin and promotes abnormalities in the skin barrier. It has also been shown to affect the expression of genes in keratinocytes related to cell adherence, thereby increasing the risk of AD (Gutowska-Owsiak et al., 2012).

In the present study, 13 prospective hub genes were screened using STRING and the cytoHubba plugin in Cytoscape. We found nine hub genes (CCR7, CCNA2, CXCL10, IRF7, ISG15, MKI67, MMP1, NCAPG, and RRM2) with high diagnostic sensitivity and specificity by external dataset GSE120721 validation and ROC curve analysis. For experimental validation, we performed immunohistochemistry analysis for 12 individuals, including 6 AD patients and 6 age-matched, race-matched and sex-matched healthy individuals, to detect expression levels of these nine identified hub genes. The expression levels of CCR7, CXCL10, IRF7, MMP1, and RRM2 were found to be upregulated in AD patients, as compared to the levels observed in HCs. This was in accordance with the results obtained using bioinformatics analysis. Expression levels of CCNA2, ISG15, MKI67, and NCAPG did not differ significantly in AD patients and HCs, though AD patients had higher expression levels of all four genes. We hypothesize that the non-significance of these differences could be attributed to the small sample size. This was because a greater number of skin samples could not be obtained from AD patients due to ethical reasons. Moreover, we only performed immunohistochemistry analysis. We could not simultaneously investigate protein expression levels via western blotting because of the small amount of skin tissue collected.

In this study, we identified CCR7, CXCL10, IRF7, MMP1, and RRM2 to be potential diagnostic biomarkers for AD. The chemokine receptor CCR7 was reportedly present on DCs as well as naïve, regulatory, and memory T cells (Förster et al., 2008). The movement of skin DCs to lymphoid tissue has been shown to be conditionally dependent upon the activation of the chemokine receptor CCR7 under both stable and inflammatory conditions (Ohl et al., 2004). After CCR7^−/−^ mouse bone marrow-derived DCs were subcutaneously injected into wild-type mice, these cells were unable to travel to the lymph nodes and access lymphatic drainage, which indicates that DCs also need to express CCR7 to facilitate their homing to lymph nodes (Hintzen et al., 2006). Moreover, it has been reported that the movement of neutrophils from skin to skin-draining lymph nodes via lymphatic vessels was also mediated by CCR7 (Özcan et al., 2022). In the present study, we found that the CCR7 expression level was upregulated in AD patients. As AD is a complex disease involving various immune cells such as DCs and neutrophils (Novak, 2012; Dhingra et al., 2013), it could be speculated that CCR7 might mediate the movement of DCs and neutrophils to skin-draining lymph nodes in AD and induce subsequent immune responses.

CXCL10, a 10 kDa protein classified as a Th1-chemokine, binds to CXCR3 receptors. CXCL10 exhibits chemotactic activity toward activated T lymphocytes and monocytes in the peripheral blood and is produced by activated T cells, monocytes, endothelial cells, and keratinocytes (Qi et al., 2009). In AD patients, the serum levels of CXCL10 and the expression levels of CXCL10 in skin lesions were significantly increased in comparison to the levels observed in HCs (Esaki et al., 2016; Brunner et al., 2017; Lang et al., 2021). This is consistent with our findings. CXCL10 can recruit activated T cells expressing CXCR3 (especially Th1 cells). These recruited Th1 cells secrete IFN- γ, stimulate local CXCL10 production, and further facilitate Th1 cell recruitment (Colvin et al., 2004). As T lymphocytes play vital roles in AD pathogenesis (Sroka-Tomaszewska and Trzeciak, 2021), lymphocyte chemotaxis regulated by CXCL10 may be involved in the AD immune response.

IFN regulatory factor 7 (IRF7) belongs to the IFN regulatory transcription factor family, which plays a vital role in the control of several biological processes, including inflammation, apoptosis, and immune response generation. He *et al.* found that the expression of IRF7 is increased in type 2 lymphoid cells during allergic inflammation, whereas an IRF7 deficiency leads to its remission (He et al., 2019). Cohen *et al.* reported that the phenotypic transformation from pro-inflammatory macrophages to anti-inflammatory macrophages (M1-to-M2) is modulated by IRF7, which is down-regulated by the transforming growth factor-beta 1 pathway (Cohen et al., 2014).

MMP-1, an interstitial collagenase, cleaves type I and II collagen, which are major constituents of the dermis (Parks et al., 2004). It has been shown that the serum levels of MMP-1 were significantly higher in the AD group than in HCs, and may correlate with the degree of damage to the epidermal barrier, which is represented by the extent of trans-epidermal water loss (Basałygo et al., 2021). Harper *et al.* reported that an increase in MMP-1 levels was associated with a corresponding increase in the severity of AD (Harper et al., 2010). Another report demonstrated that the severity of AD was proportional to the severity of damage to the epidermal barrier (Weidinger and Novak, 2016). MMP-1 also triggers other MMPs, such as MMP-9, which is highly specific for substrates such as dermal elastin and fibrillin (Tsoureli-Nikita et al., 2006). The cleavage of the components in the basement membrane allows T cells to cross the basement membrane and enter the epidermal compartment during skin inflammation (Wölfle et al., 2015).

RRM2, a small subunit of ribonucleotide reductase, is overexpressed by tumors, and plays a role in resistance to chemotherapy (Zhan et al., 2021). Tang *et al.* reported that there was a positive correlation between the expression level of RRM2 and the extent of infiltration by neutrophils and macrophages. They also reported that RRM2 inhibition effectively suppressed macrophage infiltration, and affected the balance of macrophage polarization, which promoted M1 phenotype polarization and suppressed the M2 phenotype *in vitro* and *in vivo* (Tang et al., 2021). However, there have been no reports about the relationship between RRM2 and AD until now. We found that RRM2 was upregulated in AD skin tissue. This may contribute to the chronic inflammation involved in AD, since RRM2 is known to promote macrophage infiltration and polarization. However, this hypothesis needs to be validated by further studies.

Immune cell infiltration is a hallmark of AD. Therefore, we studied infiltration of immune cells, and relationships between the identified hub genes (CCR7, CXCL10, IRF7, MMP1, and RRM2) and significant differential immune cells (naive CD4^+^ T cells, plasma cells, activated DCs, and resting mast cells) in AD. We found that CCR7 expression level was positively correlated with the number of CD4^+^ naïve T cells (R = 0.42, *p* = 0.011) in AD patients. It has been shown that CCR7 mediates the homing of naive T cells. Studies in CCR7-deficient (CCR7^−/−^) mice have demonstrated that on examination of lymph nodes and Peyer’s patches, naive T cell numbers are reduced. When the T cells of CCR7^−/−^ mice were adoptively transferred to wild-type mice, they were unable to home to Peyer’s patches and draining lymph nodes (Förster et al., 1999). We hypothesized that CCR7 might also mediate the homing of naive T cells in AD. However, more *in vitro* and *in vivo* studies need to be conducted to validate this hypothesis.

Several limitations are associated with our study. First, the human sample size was limited. Additional samples are required to validate the present findings. Second, we used only immunohistochemistry techniques to validate hub genes *in vitro*. Additional experiments with more quantitative methodologies will be needed for further confirmation of our results. Finally, the precise role of identified hub genes in AD requires additional elucidation both *in vitro* and *in vivo.* Further research is essential to determine whether CCR7, CXCL10, IRF7, MMP1, and RRM2 could be used as predictive biomarkers for the diagnosis and treatment of AD.

## Conclusion

To summarize, our study uses both WGCNA and CIBERSORT methods to understand the molecular mechanism of AD, and to verify the result by immunohistochemical technology. Our study has not only identified effective biomarkers for AD but has increased our understanding of how immune cells related to AD. CCR7, CXCL10, IRF7, MMP1, and RRM2 could be used as biomarkers in AD for both diagnostic and therapeutic purposes. Additionally, we have shown that CCR7 expression level was positively correlated with the number of CD4^+^ naïve T cells. We therefore have confidence that our study supports a new trajectory for investigation into the management of AD and provides new knowledge on the immune, cellular, and molecular mechanisms for its pathogenesis.

## Data Availability

Publicly available datasets were analyzed in this study. This data can be found here: https://www.ncbi.nlm.nih.gov/geo/, accession numbers: GSE6012, GSE32924, GSE36842, and GSE120721.
