# Supplementary material for: Identification of Effective Diagnostic Biomarkers and Immune Cell Infiltration in Atopic Dermatitis by Comprehensive Bioinformatics Analysis
Source: Front Mol Biosci. 2022 Jul 14;9:917077. doi: 10.3389/fmolb.2022.917077 (PMC9330059; doi:10.3389/fmolb.2022.917077)
Supplement: Supplementary file 2 [file Table1.DOCX]

**Supplementary Table S1.** Characteristics of patients involved in IHC validation

| **Sample_ID** | **Gender** | **Age (years)** | **Ancestry** | **Diagnosis** | **Tissue** | **Age at diagnosis (years)** | **Treatment** |
| --- | --- | --- | --- | --- | --- | --- | --- |
| HC1 | Male | 26 | Yellow | Control | Whole Skin | NA | NA |
| HC2 | Male | 35 | Yellow | Control | Whole Skin | NA | NA |
| HC3 | Male | 21 | Yellow | Control | Whole Skin | NA | NA |
| HC4 | Female | 24 | Yellow | Control | Whole Skin | NA | NA |
| HC5 | Male | 20 | Yellow | Control | Whole Skin | NA | NA |
| HC6 | Female | 21 | Yellow | Control | Whole Skin | NA | NA |
| AD1 | Male | 26 | Yellow | AD | Whole Skin | 26 | NA |
| AD2 | Male | 35 | Yellow | AD | Whole Skin | 34 | NA |
| AD3 | Male | 21 | Yellow | AD | Whole Skin | 14 | Glucocorticoids |
| AD4 | Female | 24 | Yellow | AD | Whole Skin | 23 | NA |
| AD5 | Male | 30 | Yellow | AD | Whole Skin | 29 | NA |
| AD6 | Female | 21 | Yellow | AD | Whole Skin | 21 | NA |
