# Supplementary figures and images for: Identification of Effective Diagnostic Biomarkers and Immune Cell Infiltration in Atopic Dermatitis by Comprehensive Bioinformatics Analysis
Source: Front Mol Biosci. 2022 Jul 14;9:917077. doi: 10.3389/fmolb.2022.917077 (PMC9330059; doi:10.3389/fmolb.2022.917077)

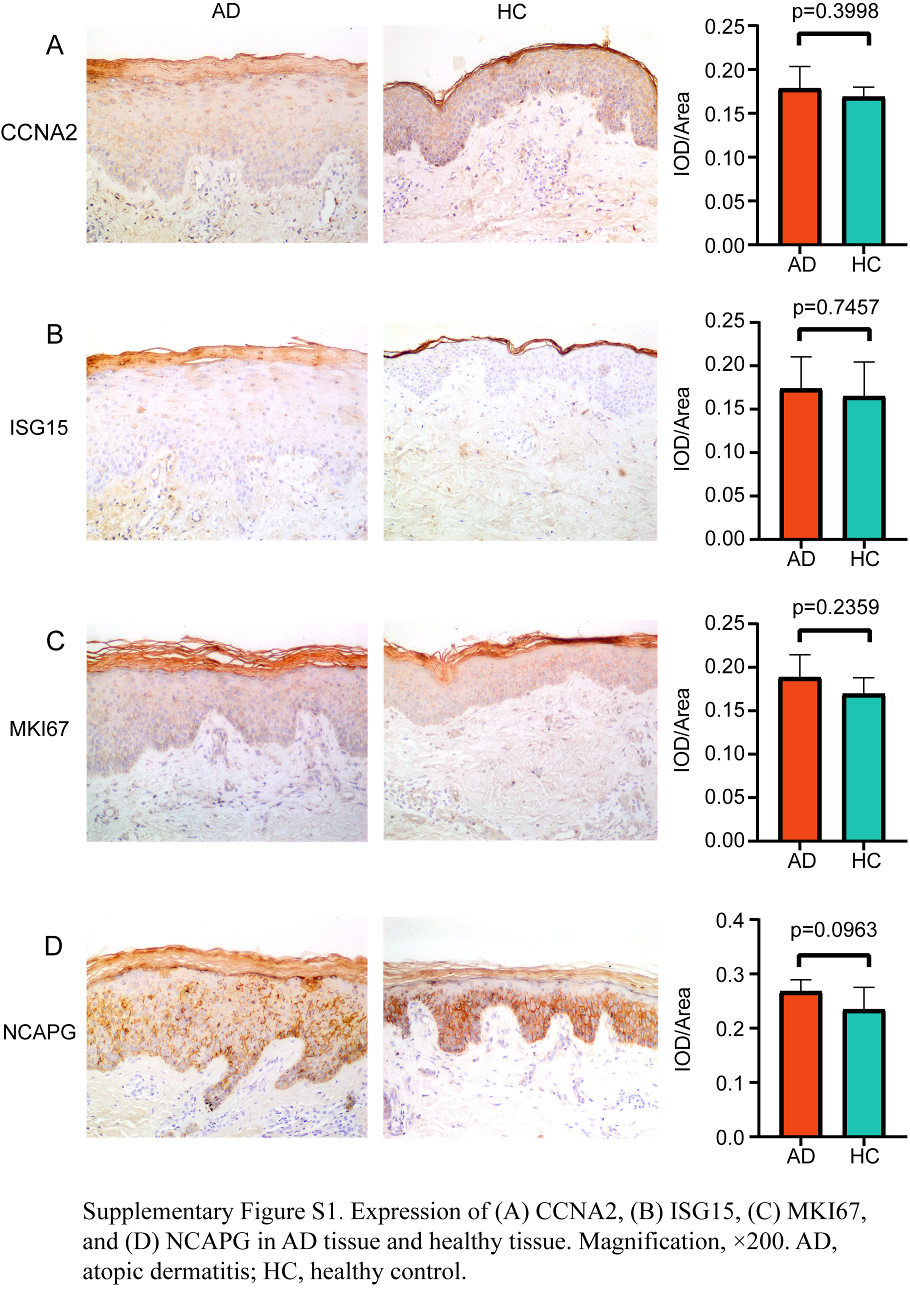

Supplement: Supplementary file 3 [file Image1.TIF]
